# All-optical analog comparator

**DOI:** 10.1038/srep31903

**Published:** 2016-08-23

**Authors:** Pu Li, Xiaogang Yi, Xianglian Liu, Dongliang Zhao, Yongpeng Zhao, Yuncai Wang

**Affiliations:** 1 Key Laboratory of Advanced Transducers and Intelligent Control System, Ministry of Education of China, Taiyuan 030024, China; 2 Institute of Optoelectronic Engineering, Taiyuan University of Technology, Taiyuan 030024, China; 3 Fiber Optical Components & Instruments Business Unit, LUSTER LightTech Co., Ltd., Beijing 100095, China; 4 School of Electronic and Information Engineering, Beijing Jiaotong University, Beijing 100044, China

## Abstract

An analog comparator is one of the core units in all-optical analog-to-digital conversion (AO-ADC) systems, which digitizes different amplitude levels into two levels of logical ‘1’ or ‘0’ by comparing with a defined decision threshold. Although various outstanding photonic ADC approaches have been reported, almost all of them necessitate an *electrical* comparator to carry out this binarization. The use of an electrical comparator is in contradiction to the aim of developing all-optical devices. In this work, we propose a new concept of an *all-optical* analog comparator and numerically demonstrate an implementation based on a quarter-wavelength-shifted distributed feedback laser diode (QWS DFB-LD) with multiple quantum well (MQW) structures. Our results show that the all-optical comparator is very well suited for true AO-ADCs, enabling the whole digital conversion from an analog optical signal (continuous-time signal or discrete pulse signal) to a binary representation totally in the optical domain. In particular, this all-optical analog comparator possesses a low threshold power (several mW), high extinction ratio (up to 40 dB), fast operation rate (of the order of tens of Gb/s) and a step-like transfer function.

All-optical analog-to-digital conversion (AO-ADC) has a significant impact on future high speed optical communication and network systems, which hope to convert a continuous-time signal to a digital binary signal entirely in the optical domain. Analog comparators are critical units in AO-ADC systems, which digitize varying amplitude levels into two levels of logical ‘1’ or ‘0’ by comparison with a defined decision threshold.

There have been many photonic ADC methods have been reported^1^. For instance, Mach-Zehnder modulators (MZMs) have been widely used to achieve photonic ADCs^2^,^3^,^4^,^5^,^6^,^7^,^8^ since Taylor firstly employed them for optical ADC^9^. The Sagnac interferometer or nonlinear optical loop mirror (NOLM) is another widely used configuration utilizing cross-phase modulation (XPM) in highly nonlinear fibers (HNLFs) for ADC^10^,^11^,^12^,^13^. Some other optical ADC methods have also been extensively demonstrated using spectral frequency shift^14^,^15^,^16^,^17^ or slicing the supercontinuum spectrum^18^,^19^. However, their response functions are sinusoidal-like which induce a large decision ambiguity between the two expected digitized levels (i.e., logical ‘0’ and ‘1’). Therefore, electrical analog comparators have to be cascaded at the outputs so as to provide two distinct levels. Besides, a relatively large input peak power is usually necessary to generate sufficient nonlinear effects for the photonic ADC based on standard optical media. When standard optical media are replaced by special nonlinear materials, significant nonlinear behaviors can be manifested at a low power level, but their specialized processing is not beneficial for practical applications. In a word, it is still far from thorough AO-ADCs due to the lack of efficient all-optical comparator technologies, though various outstanding photonic ADC schemes have been proposed. The requirement of electronic comparators, confronted with the ‘electrical bottleneck’, deviates from the original intention to develop ultra-fast all-optical techniques.

In this paper, we propose a new concept of ‘all-optical’ analog comparator which does the aforementioned binarization in the optical domain. We numerically demonstrate an implementation method based on optical hysteresis. Specifically, we present a proof-of-concept numerical demonstration of an all-optical analog comparator based on a quarter-wavelength-shifted distributed feedback laser diode (QWS DFB-LD) with multiple quantum well (MQW) structures and antireflection (AR)-coated facets. Our simulations demonstrate that the QWS DFB-LD exhibits excellent comparator performances, such as a step-like transfer function, large extinction ratio (*ER*) of 40 dB, low threshold power (several mW) and fast operating rate at several tens of GHz. Herein, ‘threshold power’ corresponds to the input peak power, not the average power. In addition, further simulations show that our all-analog comparator can also be extended to the realization for all-optical multi-bit ADC, although it is not the main point of the present work.

Compared to the widely applied electrical analog comparator in photonic ADCs, the all-optical analog comparator can overcome the ‘electrical bottleneck’ and has the capability of being compatible with future all-optical communication and network systems, with no need of E/O or O/E conversion. Therefore, it is expected that our work may remove the current obstacle of electrical analog comparator and thereby assist with the realization of true AO-ADCs.

## Results

**Figure 1:**
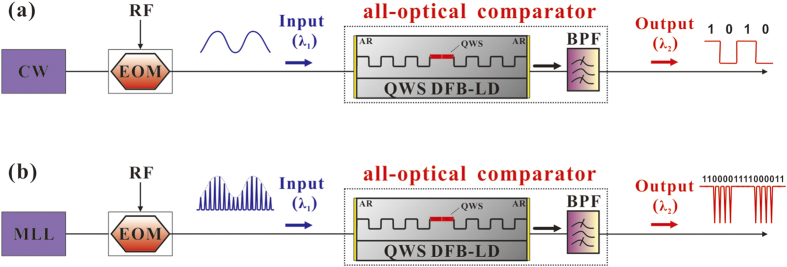
Schematic diagram for demonstrating the performance of the all-optical comparator. (**a**) Continuous-time scenario; (**b**) Discrete-time scenario. CW, Continuous-wave laser diode; MLL, Mode-locked laser; RF, Sinusoidal RF signal; EOM, Electro-optic modulator; QWS, Quarter-wavelength-shift; AR, antireflection (AR)-coated facets; BPF, Band-pass filter.

**Figure 2:**
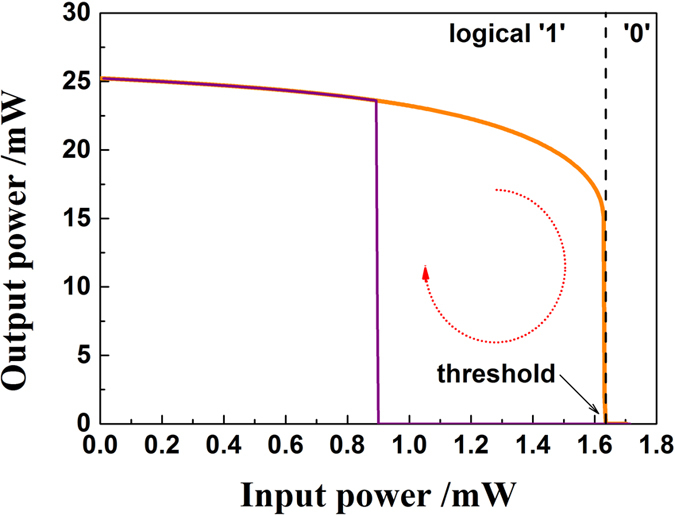
Transfer function of the all-optical analog comparator under the operational point (*L* = 600 μm, *κ* = 20 cm^−1^, *r* = 10^−4^ and *I* = 4*I*_th_).

As shown in the dash box in Fig. 1, the all-optical analog comparator consists of a multiple quantum well (MQW) QWS DFB-LD with AR-coated facets and a band-pass filter (BPF). The BPF is used to filter out the external injection light and spontaneous emission noise and only transmit the lasing signal of the QWS DFB-LD. The most important feature of the QWS DFB-LD is the π/2 phase shift at the center of its DFB region corresponding to a quarter of a wavelength in the waveguide. When the QWS DFB-LD is injected by an external light with a wavelength *λ*_1_, an optical hysteresis can arise between the power of input external light and the output power of the QWS DFB-LD with a lasing wavelength *λ*_2_. Such an optical hysteresis arises due to the spatial-hole-burning (SHB) effect induced by nonlinear amplification of the external input light. Figure 2 shows a typical hysteresis: a bistable response of the QWS DFB-LD under a continuous-wave (CW) optical injection. One output state (logical ‘1’) corresponds to the QWS DFB-LD lasing, while the other state (logical ‘0’) corresponds to the laser being turned off. From it, we can see that the transfer function between the input and the output is close to a rectangle and has a very steep threshold around a low input power of 1.64 mW. Moreover, the extinction ratio (*ER*) is calculated as *ER* (dB) = 10 lg (*P*^1^_min_/*P*^0^_max_) = 40 dB, where *P*^1^_min_ and *P*^0^_max_ are the minimum and maximum output power of the logical ‘1’ and ‘0’ regions, respectively. In our simulations, the QWS DFB-LD is biased at four times its threshold current (*i.e*., *I* = 4*I*_th_ = 104 mA), which has a chip length *L* = 600 μm, grating coupling coefficient *κ* = 20 cm^−1^ and facet reflectivity *r* = 10^−4^. We note that in simulations there are no strict requirements for the wavelength of the injected light (*λ*_1_), except that it should be outside the QWS DFB-LD stop-band (approximately 3 nm wide) to avoid interaction with the DFB grating. Herein, *λ*_1_ = 1550 nm and *λ*_2_ = 1560 nm are set. Detailed theoretical model and parameters are given in the last *Section* of **Methods**.

**Figure 3:**
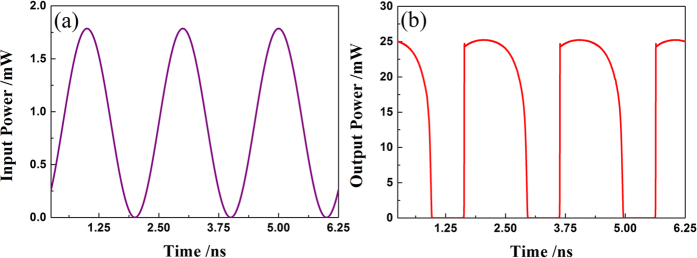
Dynamical transmission characteristic of the all-optical comparator in the case of continuous-time scenario. (**a**) Input signal waveform of a 500 MHz sine signal with a wavelength of 1550 nm; (**b**) Output signal waveform of the all-optical comparator with a wavelength of 1560 nm.

**Figure 4:**
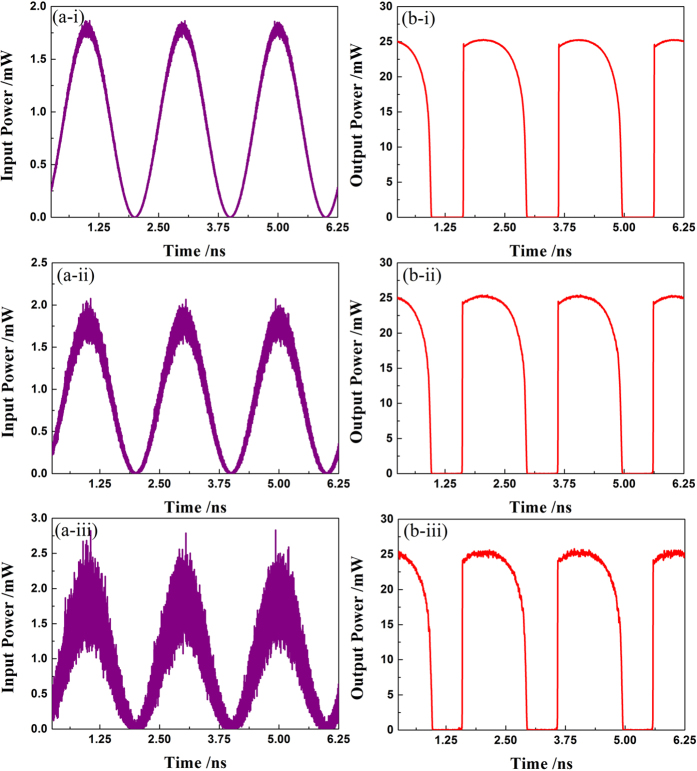
Anti-noise jamming analysis of the all-optical comparator for the continuous-time scenario. (**a-i**), (**a-ii**) and (**a-iii**) are the input signal waveforms of the 1550 nm sine signal with different signal-noise ratio (SNR) of 35 dB, 25 dB and 15 dB, respectively; (**b-i**), (**b-ii**) and (**b-iii**) are the associated output signal waveforms of the 1560 nm all-optical comparator, respectively.

Figure 1(a,b) are schematic diagrams demonstrating the performance of the all-optical analog comparator when it is used to digitize continuous-time and discrete-time signals, respectively. For the continuous-time scenario [Fig. 1(a)], the input signal is an analog optical signal obtained through modulating a *λ*_1_ = 1550 nm CW light with a 500 MHz sinusoidal RF signal (RF). Figure 3 shows the associated quantization results: Fig. 3(a) depicts the time series of the input signal, while Fig. 3(b) is the associated output of the lasing signal waveform of the QWS DFB-LD at *λ*_2_. Through comparing them with each other, a steep nonlinear transient effect can also be found with an extinction ratio (*ER*) about 40 dB. This is consistent with the transfer function shown in Fig. 2, except for a small transient overshoot at the rising edge. Further, the anti-noise jamming ability of the all-optical comparator is analyzed in the case of continuous-time scenario. Figure 4 gives typical input versus output plots, where the input signal is impaired by Gaussian noise at different levels. Herein, the signal-noise ratio (*SNR*) is defined as *SNR* = 10 lg (*P*_s_/*P*_n_), where *P*_s_ and *P*_n_ are the efficient power of the sinusoidal signal and the mixed noise, respectively. From Fig. 4b(i–iii), one can see that the output waveforms are nearly the same as that in Fig. 3(b), even if the *SNR* has decreased gradually from 35 dB [Fig. 4(a-i)] and 25 dB [Fig. 4(a-ii)] to 15 dB [Fig. 4(a-iii)]. This indicates that this comparator can quantize continuous-time signals with a high *ER*. Moreover, the comparator has an excellent ability of anti-noise jamming in the continuous-time case. In fact, this anti-jamming ability is caused by the positive feedback introduced by the hysteresis characteristic.

**Figure 5:**
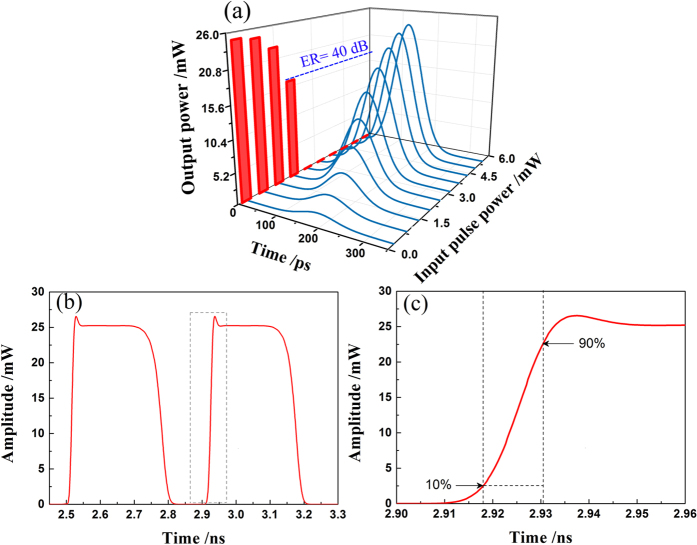
Dynamical performance of the all-optical comparator for the discrete-time scenario. (**a**) Input-output transfer function (Input pulse shapes are shown in black on the same x time axis, but with amplitude that depends on the input power in y axis. The output power corresponding to the associated input pulse is displayed in red on the left y-z plane. As input power increases through threshold, the output power jumps from high ‘1’ levels into low ‘0’ level around 0 mW.); (**b**) Output waveform corresponding to two continuous input pulses whose powers both are higher than the absolute threshold and (**c**) its associated fine structure.

For the discrete-time scenario [Fig. 1(b)], we take a single-bit ADC as an example. Actually, it is the most important application of an analog comparator in photonic ADC systems to digitize a train of optical pulses with varying amplitudes into two levels, irrespective of the ADCs are multi-bit or single-bit ADCs^1^,^2^,^3^,^4^,^5^,^6^,^7^,^8^,^9^,^10^,^11^,^12^,^13^,^14^,^15^,^16^,^17^,^18^. In our case, the optical pulse stream to be digitized is obtained through the most widely used electro-optic modulator (EOM) in photonic ADCs^2^,^3^,^4^,^5^,^6^,^7^,^8^,^9^. Figure 5 shows the results when our all-optical comparator operates in the discrete-time mode. Figure 5(a) shows the output power transfer function for a train of Gaussian optical pulses modulated by a 1.25 GHz sinusoidal RF wave via an EOM. The repetition rate, pulse width and wavelength of the input pulse train are 2.5 GHz, 100 ps and 1550 nm. The input pulse shapes are shown in black on the same x time axis, but with amplitude that depends on the input power in y axis. The output power corresponding to the associated input pulse is displayed in red on the left y-z plane. As the input power increases through a threshold, the output power jumps from high ‘1’ level into low ‘0’ level around 0 mW. One can observe that the extinction ratio (*ER*) between the output power levels of ‘1’ and ‘0’ can reach up to 40 dB, which indicates a high-quality threshold decision. Further investigations [Fig. 5(b,c)] show that the all-optical comparator possesses a high response bandwidth, which is critical for practical applications. Figure 5(b) shows a segment of the output waveform which corresponds to these input pulses with a power higher than the threshold. Through enlarging Fig. 5(b) from 2.90 ns to 2.96 ns, we can observe the fine structure of the rising edge, as shown in Fig. 5(c). According to the common definition of the rise time [The time during which the signal amplitude changes from 10% to 90% of the maximum steady state], we obtain the rise time of our comparator to be about 12.5 ps, which corresponds to a 3-dB bandwidth of 28 GHz.

## Discussions

**Figure 6:**
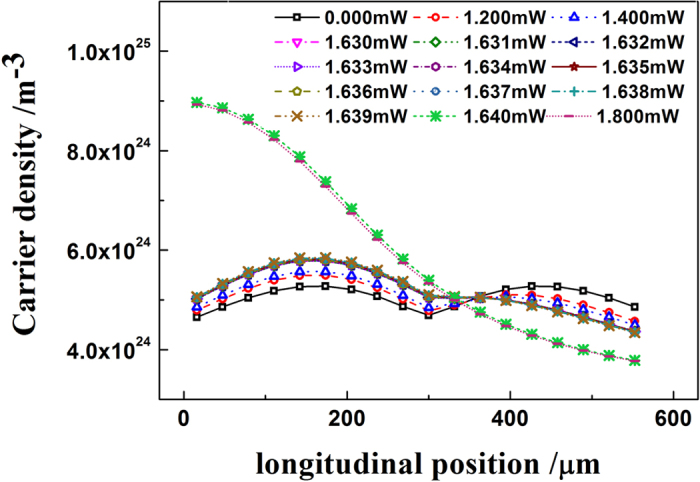
Carrier density distributions when the comparator is injected under different input levels.

The physical mechanism responsible for the step-like decision threshold transfer function of our all-optical comparator is first of all to be caused by the sudden change of carrier density distribution. Specifically, we observed the carrier density distributions along the chip length when our comparator works at different input power levels around the transition region between the logical ‘1’ and logical ‘0’ region. From Fig. 2, one can see the transition region is near the threshold power of 1.640 mW. The associated carrier density distributions when the input power varies from 1.630 to 1.640 mW (near its transition region) with a step of 0.001 mW is shown in Fig. 6. For comparison, we also give the carrier density distributions when the comparator works at several typical points in the logical ‘1’ region [Input power = 0 mW, 1.2 mW or 1.4 mW] and low-level region [Input power = 1.8 mW]. We find that the laser has a relatively uniform carrier density distribution when the input power varies from 0 mW to 1.639 mW. In this region, only a small change of the carrier distribution is observed: the carrier density in the first half of the laser length slightly increases and in the latter part decreases with input power enhanced. This small change in carrier density distribution induces that the lasing power slowly decreases with the increase of input power. However, it must be noted that this slight change is not enough to extinguish the laser. Until the input power reaches above 1.640 mW, the laser will abruptly switch into the non-lasing state [logical ‘0’ region]. At this time, we can see that the associated carrier density becomes extremely non-uniform. That is to say, the carrier density monotonically decreases along the laser length and thus the laser cannot remain in a lasing state. This demonstrates an extremely vertical transition region – the output ‘jumps’ between ‘0’ and ‘1’ when the input changes by as little as 0.001 mW(=1.640 − 1.639 mW).

Secondly, we discuss the flatness of the logical ‘1’ of our comparator. The flatness can be defined as *β* (dB) = 10 log (*P*^1^_max_/*P*^1^_min_), where *P*^1^_max_ and *P*^1^_min_ are the maximum and minimum output power of the logical ‘1’ region, respectively. As shown in Fig. 2, the output of logical ‘1’ in our comparator is not a constant, although the *ER* between ‘0’ and ‘1’ levels is larger than 40 dB. This fluctuation in logical level ‘1’ is called 1-level ‘noise’. Strictly speaking, it is not beneficial for a high-quality all-optical comparator and should be eliminated. A simple method to remove this imperfection is to introduce a CW light with the same wavelength as the input signal at the input port of the comparator. However, it must be guaranteed that the power of the CW light must not be above the front edge of the bistable region [Fig. 2]. Simultaneously, the input signal should be attenuated appropriately. The closer the CW optical power stays to the front edge of the bistable region, the less *P*^1^_max_ will be. Accordingly, the flatness *β* = 10 log (*P*^1^_max_/*P*^1^_min_) gets improved.

Thirdly, we evaluate the feasibility of manufacturing the specially designed phase-shift DFB laser which is the core unit of the all-optical analog comparator. Although the QWS DFB-LD uses a non-uniform grating with a quarter-wave phase shift (QWS) in the middle, which is different from the normal DFB with uniform grating structures, the device still can be manufactured using standard DFB manufacturing technologies. Specifically, the non-uniform grating can be fabricated by electron beam lithography (EBL) to form a quarter-wave phase shift (QWS) ref. [20]. Moreover, it also can be fabricated by equivalent phase shift technique for easy and fast fabrication refs [21,22]. The laser epi-wafer can be fabricated by the conventional two-stage lower-pressure metal-organic vapor phase epitaxy (MOVPE) process. First, AlGaInAs (or InGaAsP) compressively strained multiple-quantum-well (MQW) as the active region is sandwiched between two separate confinement heterostructure (SCH) layers. The grating is then fabricated on the up-SCH layer. Afterwards, a p-type InP cladding layer and a p-InGaAs contact layer are re-grown. The post-processes to fabricate the waveguide, electrodes and facet coatings are also the same as that of the normal DFB lasers ref. [23]. In addition, we note that the phase-shifted DFB-SOA in refs [24, 25] apply a very similar structure of the phase-shifted DFB-LD.

**Figure 7:**
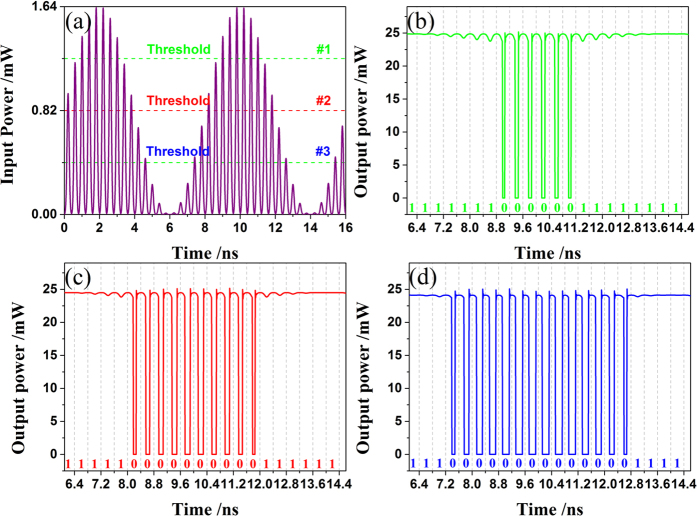
Simulation results for *m*-bit ADC based on 2^m^ − 1 all-optical comparators. Herein, take *m* = 2 as an example. (**a**) Time-series of the Input pulse signals to be quantized from 0 to 16 ns. (**b**–**d**) are the associated output waveform of all-optical comparator #1, #2 and #3 from 6.1 to 14.6 ns, respectively. The binary output states are depicted in the strip below the waveform traces in (**b**–**d**).

Finally, we point out that our all-analog comparator can also be extended to the realization of all-optical multi-bit ADCs, although this is not the focus of our work. Following the method for implementing electrical *m*-bit ADCs, we can achieve an all-optical *m*-bit ADC through equally dividing the input signal into 2^m^ quantization levels by means of 2^m^ − 1 all-optical analog comparators with different thresholds. For conciseness, we take a 2-bit ADC (*i.e*., *m* = 2) as an example. As shown in Fig. 7(a), 3 all-optical analog comparators (#1, #2 and #3) are needed to set 3 different threshold levels, which divide the whole input power scope to 4 equal parts. Then, each part from top to bottom can be coded into **00**, **01**, **10** and **11**, respectively. These 2-bit binary codes correspond to a set of comparator output states, as shown in Table 1.

**Figure 8:**
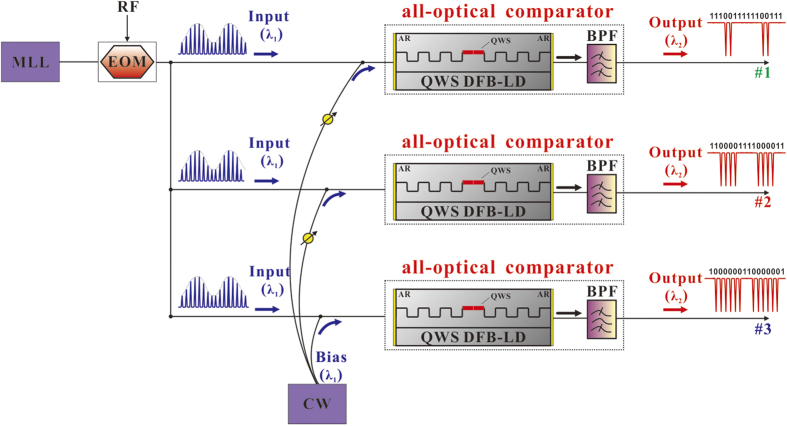
Schematic diagram for demonstrating the performance of the all-optical comparator. CW, Continuous-wave laser diode; MLL, Mode-locked laser; RF, Sinusoidal RF signal; EOM, Electro-optic modulator; QWS, Quarter-wavelength-shift; AR, antireflection (AR)-coated facets; BPF, Band-pass filter.

Figure 8 is a scheme for 2-bit ADC. Three identical input analog pulse signals can be obtained by equally dividing the EOM output. Through appropriately selecting the intensities of the MLL and RF signal, we can ensure that the three input pulse signals have the same waveform as Fig. 7(a), where the average value of all the pulse peaks is calculated to be 0.82 mW. Then, each input analog pulse signal will be quantized separately by three all-optical comparators whose transfer functions are the same as Fig. 2. Considering the three all-optical analog comparators have the same inherent threshold at 1.64 mW [Fig. 2], we then introduce three different biases to obtain the three equivalent thresholds (#1, #2 and #3) marked in Fig. 7(a). In practice, the biases actually can be supplied by a continuous-wave (CW) laser diode with the same wavelength as the input signals. By controlling the power of the CW output and tuning the variable optical attenuators (VOA), one can accurately set the three different biases (#1, #2 and #3) at 0.41 (=1.64 × 1/4) mW, 0.82 (=1.64 × 2/4) mW and 1.23 (=1.64 × 3/4) mW, respectively. Accordingly, the three equivalent thresholds of all-optical comparators (#1, #2 and #3) will be 1.23 mW, 0.82 mW and 0.41 mW, respectively. Figure 7 gives the associated simulation results for a 2-bit ADC. Fig. 7(a) is the input analog signal, which is a train of Gaussian optical pulses modulated by a 0.125 GHz sinusoidal RF wave via an electro-optic modulator (EOM). The repetition rate, pulse width and wavelength of the input pulse train are 2.5 GHz, 100 ps and 1550 nm. Figure 7(b–d) show the output times-series of the all-optical comparator #1, #2 and #3, respectively. The other parameters are the same as that in Table 1. Through comparing the input time-series from 6.1 to 14.4 ns with the corresponding three comparator outputs, it is clear that our results are consistent with the expectation in Table 1. This means that a multi-bit ADC can be implemented using our method.

## Methods

### Theoretical Model

Simulations of the architecture in Fig. 1 are carried out utilizing a physically accurate simulation environment for photonic systems (VPIcomponentMaker™Photonic Circuits from VPI photonics)^26^. The simulator for the QWS DFB-LD is based on the Transmission-Line Laser Model (TLLM), which is a full travelling-wave (TW) model. It was shown recently in several publications refs [27, 28, 29] that this program can be used to describe an optically-injected system accurately as well as to investigate the internal properties. Generally, the TLLM uses a time-domain model of the DFB-LD to model the changing carrier and photon densities along the DFB-LD length by dividing the cavity into a number of longitudinal subsections. Each subsection consists of traveling-wave equations for the forward and backward traveling optical fields [denoted as *A*^+^ (*j*) and *A*^−^ (*j*) in the following] and models for gain, spontaneous emission, carrier-dependent index and loss. Each subsection also contains a rate equation for the carrier density, which is fed by the injection current, but depleted by spontaneous recombination and stimulated recombination calculated from the square of the sum of the forward and backward traveling optical fields. The optical fields and carrier density in each of these subsections are simulated separately and then their obtained results are passed to adjacent sections through a transmission matrix where a new calculation step starts.

For the uniform grating subsection *j*, the transmission matrix ***T*** can be derived from these traveling-wave equations as below:


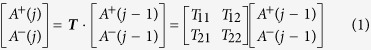



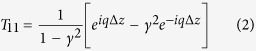



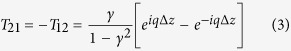



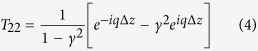


where Δ*z* represents the length of subsection and *γ* is the effective reflectivity induced by distributed feedback and can be indicated as follows:


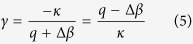


where *κ* is the grating coupling coefficient, Δ*β* is a measure of the detuning of the laser mode from the Bragg wavelength, and *q* equals to ± (Δ*β*^2^ − *κ*^2^)^1/2^.

As for the π/2 phase-shifted subsection, the transmission matrix ***T*** should be multiplied by a matrix ***T***_**p**_:


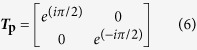


In this way, the non-uniform distribution of the optical field and the carrier density can be handled. A more detailed explanation of this method is described in the literature ref. [30]. It must be mentioned that the simulated QWS DFB-LD with external optical injection in our work employs a multiple quantum well (MQW) active region. The parameters used in simulation can be found in Table 2.

## Additional Information

**How to cite this article**: Li, P. *et al*. All-optical analog comparator. *Sci. Rep*. **6**, 31903; doi: 10.1038/srep31903 (2016).

## Figures and Tables

**Table 1:** Code lookup table.

Comparator output state			2-bit binary code state	
#1	#2	#3	d_1_	d_0_
0	0	0	0	0
1	0	0	0	1
1	1	0	1	0
1	1	1	1	1

**Table 2:** Simulation Parameters of the QWS DFB-LD.

Parameter	Value	Unit
Nominal wavelength	1560	nm
Threshold Current (*I*_th_)	26	mA
Laser Chip Length	600	μm
Bias Current	104	mA
Active Region Width	2.5	μm
Active Region Thickness (MQW)	0.04	μm
SCH Region Thickness	0.21	μm
Facet Reflectivity	10^−4^	
Grating Coupling Coefficient	20	cm^−1^
Group Effective Index	3.7	
Internal Loss	30	cm^−1^
Linear Recombination Coefficient	1 × 10^9^	s^−1^
Bimolecular Recombination Coefficient	5 × 10^−15^	m^3^ s^−1^
Auger Recombination Coefficient	6 × 10^−40^	m^6^ s^−1^
Linear Material Gain Coefficient	3 × 10^−20^	m^2^
MQW Confinement Factor	0.07	
SCH Confinement Factor	0.56	
Transparency Carrier Density	1.5 × 10^24^	m^−3^
Carrier Capture Timeconstant	1 × 10^−12^	s
Carrier Escape Timeconstant	2 × 10^−12^	s
